# A novel mutant allele of *AtCNGC15* reveals a dual function of nuclear calcium release in the root meristem

**DOI:** 10.1093/jxb/erad041

**Published:** 2023-01-30

**Authors:** Emily Tipper, Nuno Leitão, Pierre Dangeville, David M Lawson, Myriam Charpentier

**Affiliations:** Department of Cell and Developmental Biology, John Innes Centre, Colney Lane, Norwich NR4 7UH, UK; Department of Cell and Developmental Biology, John Innes Centre, Colney Lane, Norwich NR4 7UH, UK; Department of Cell and Developmental Biology, John Innes Centre, Colney Lane, Norwich NR4 7UH, UK; Department of Biological Chemistry, John Innes Centre, Colney Lane, Norwich NR4 7UH, UK; Department of Cell and Developmental Biology, John Innes Centre, Colney Lane, Norwich NR4 7UH, UK; University of Warwick, UK

**Keywords:** Arabidopsis, calcium signalling, cyclic nucleotide gated channel, lateral organ boundaries domain, nitrate, root development

## Abstract

Calcium release to the nucleoplasm of root meristem cells was demonstrated to modulate root development. The calcium channel encoded by cyclic nucleotide-gated channel (CNGC) 15 localizes at the nuclear envelope in young Arabidopsis seedlings. In contrast, at later stages of root growth, overexpression analysis showed that AtCNGC15 can relocalize to the plasma membrane to mediate primary nitrate-induced gene expression. This raises the question as to whether nuclear localized AtCNGC15 is required for root apical meristem development in young Arabidopsis seedlings, and whether nitrate signalling occurs independently of nuclear localized AtCNGC15 at this developmental stage. In this study, we characterize a novel mutant allele of *AtCNGC15* and demonstrate that the mutation of a highly conserved aspartic acid in the C-linker domain is sufficient to impair the gating of AtCNCG15. We demonstrate that AtCNGC15 mediates the nuclear calcium release that modulates root apical meristem development and nitrate-induced *LBD39* expression. We also show that, in the presence of nitrate, the relocalization of AtCNGC15 at the plasma membrane occurs specifically in the columella cells. Our results further suggest that the induction of *LBD37*, *LBD38*, and *LBD39* in the presence of nitrate is modulated by different inputs of cytoplasmic or nuclear calcium release.

## Teaser

Nuclear calcium release, mediated by cyclic nucleotide-gated channel 15, modulates root meristem development and nitrate-induced gene expression in Arabidopsis primary root meristems.

## Introduction

Plant cyclic nucleotide-gated channels (CNGCs) are central to calcium signalling in myriad processes, including development and biotic and abiotic stress response (Jarratt-Barnham *et al.*, 2021). In recent years, advances in their characterization have revealed that the modular architecture of the CNGCs, which assemble as homo- and/or hetero-tetramers, contributes to their plurality of function and mechanisms of regulation (Pan *et al.*, 2019; Yu *et al.*, 2019). In plants, each CNGC subunit consists of six membrane-spanning segments designated S1 to S6, followed by a cyclic nucleotide-binding domain (CNBD) and a calmodulin-binding domain near the C-terminus (Kaplan *et al.*, 2007). In addition, a stretch of amino acids called the C-linker connects the CNBD to the S6. Although the function of the C-linker has not been explored in plant CNGCs, it plays an essential role in coupling ligand binding to pore opening in mammalian cyclic nucleotide-gated (CNG) channels (Mazzolini *et al.*, 2018), a gating mechanism that is influenced by the subunit composition of the tetramer (Zheng *et al.*, 2022).

CNGCs mediate calcium release in various plant cell types and cellular compartments (Dietrich *et al.*, 2020; Jarratt-Barnham *et al.*, 2021). In the legume *Medicago truncatula*, three nuclear-localized CNGC15 paralogues, CNGC15a, CNGC15b, and CNGC15c, in complex with the ion channel Does not Make Infections 1 (DMI1), are required for the generation of nuclear calcium oscillations that are essential for the establishment of nitrogen-fixing bacterial and arbuscular mycorrhizal symbioses (Charpentier *et al.*, 2016). The C-termini of the three MtCNGC15s associate with holo-Calmodulin 2, which modulates their activities by providing the negative feedback to close the CNGC15 channel (Del Cerro *et al.*, 2022). Interestingly, the three CNGC15s have different affinities for holo-Calmodulin 2, suggesting that they could form a heterocomplex to regulate nuclear calcium oscillation (Del Cerro *et al.*, 2022). In Arabidopsis, which cannot perform symbioses with arbuscular mycorrhizal or nitrogen-fixing bacteria, the functional orthologue of MtDMI1 is encoded by *AtDMI1* and modulates the nuclear calcium release associated with primary root apical meristem development and auxin homeostasis (Leitão *et al.*, 2019). In contrast to *M. truncatula*, only one *AtCNGC15* is present in the Arabidopsis genome (Maser *et al.*, 2001). Expression of *AtCNGC15* complements the symbiosis defect of the double mutant *MtCNGC15c,b* (Leitão *et al.*, 2019), and AtCNGC15 localizes at the nuclear envelope in close proximity to AtDMI1 at the early stages of root development (6 d) (Leitão *et al.*, 2019; Wang *et al.*, 2021). However, whether AtCNGC15 contributes to the nuclear calcium release associated with the cellular development of the root apical meristem in young seedlings remains to be demonstrated. It was recently shown that at a later stage (12 d) of root growth, AtCNGC15 can form a complex with the plasma membrane nitrate transceptor NRT1.1 to regulate nitrate signalling (Wang *et al.*, 2021). As AtCNCG15 localizes to the nuclear envelope in young roots, this observation suggests that nitrate signalling would occur independently of AtCNGC15.

Herein, we use a combination of genetics, cell imaging, electrophysiology, and gene expression analyses, and characterize a novel *Atcngc15* mutant allele to address this hypothesis. We first reveal that the C-linker region of AtCNGC15 is important for its gating. We further demonstrate that in 6-day-old roots, nuclear localized AtCNGC15 is required to modulate the nuclear calcium release associated with root meristem development and that nuclear calcium modulates the nitrate-induced expression of *LATERAL ORGAN BOUNDARIES DOMAIN* (*LBD*)*39*. We further show that AtCNCG15 relocalizes to the plasma membrane specifically in columella cells upon high nitrate treatment. Our findings demonstrate that nuclear calcium signals can influence both development and nitrate-induced gene expression and that the plurality of AtCNGC15 localization can be influenced by abiotic factors in a cell type specific manner.

## Materials and methods

### Plant material and growth conditions

Arabidopsis accessions SALK_066135C (*Atdmi1-1*), SAIL_303_C02 (*Atdmi1-2*), the Arabidopsis TILLING line 12379 (*Atcngc15*^*D408N*^), and wild-types Col-0 and Col-0 *er-105* were purchased from the Nottingham Arabidopsis Stock Centre. The *M. truncatula cngc15b-2 cngc15c-1* double mutant generated in Charpentier *et al.* (2016) was used for nodulation complementation experiments. Arabidopsis seeds were surface-sterilized in 1.2% bleach for 20 min, followed by five washes in sterile water. The seeds were plated in half-strength Murashige and Skoog (MS) medium, 1% sucrose, 0.8% agar pH 5.8 and left to stratify for 3–5 d in dark conditions at 4 °C. Plates were moved to a growth cabinet (23 °C, 16-h photoperiod, and 300 µmol m^−2^ s^−1^ light intensity). Nitrate free ½ MS medium and 10 mM nitrate ½ MS medium were made by adding 1 mM l-glutamine (Merck) and 10 mM KNO_3_, respectively, to nitrate-free ½ MS medium.

### Genotyping

Homozygosity of the *Atcngc15*^*D408N*^ allele was assessed by PCR and sequencing. A 743 bp fragment was amplified by PCR using primers 5 and 6 listed in Supplementary Table S1, and the PCR product was sequenced using primer 5 (Supplementary Table S1). The electropherogram was analysed at position +1570, where the G to A point mutation lies. Homozygosity of *Atdmi1-1* to generate the double mutant *dmi1-1cngc15*^*D408N*^ was assessed by PCR as previously described (Leitão *et al.*, 2019).

### Golden gate cloning

The construct expressing the genomic sequence of *AtCNGC15* fused to *Green Fluorescent Protein* (*GFP*) under its native promoter was generated via Golden Gate. Golden Gate cloning was conducted as outlined in Engler *et al.* (2008, 2009). The DNA fragments, flanked by orthogonal pairs of *Bsa*I recognition sites, were synthesized by Thermo Fisher Scientific. Details of assembly can be found in Supplementary Table S2.

### Generation of Arabidopsis stable transgenic lines

#### Agrobacterium

*tumefaciens*-mediated gene transfer by inflorescence infiltration (Clough and Bent, 1998) was used to generate Col-0 lines expressing *pAtCNGC15:AtCNGC15:GFP* and *pAtUBI10:Atcngc15*^*D408N*^*:GFP* as well as *cngc15*^*D408N*^ expressing NRCG-GECO1.2 (nuclear-localized red and cytoplasmic-localized green- GECO1.2, a dual-localized fluorescent calcium sensor; Kelner *et al.*, 2018). The *dmi1-1* line expressing NRCG-GECO1.2 was generated in a previous study (Leitão *et al.*, 2019). The T_1_ generation was produced by floral dipping plants after 4–5 weeks of growth. Seeds were collected and germinated under the appropriate selection in the T_1_ and T_2_ generations. The segregating population described in Supplementary Fig. S4 was generated by manual hand-pollination of *Atcngc15*^*D408N*^ with the wild-type Col-0 *er-105*. The *dmi1-1 cngc15*^*D408N*^ double mutant expressing NRCG-GECO1.2 was generated by manual hand pollination of *dmi1-1* and *cngc15*^*D408N*^ expressing NRCG-GECO1.2, followed by seed propagation and genotyping of the T_2_ generation by PCR to obtain homozygous mutants.

### Primary root length measurements

Seedlings were grown vertically on ½ MS plates. Six seedlings of the control genotype and six seedlings of the test genotype were sown per plate, and photographed at day 6 after transfer to the growth chamber. Root length was quantified using ImageJ 1.48v (NeuronJ plugin, 1.4.3v) (Meijering *et al.*, 2004).

### Characterization of the root meristem and transition zone

Fixation and staining were carried out as per Leitão *et al.* (2019). Briefly, samples were fixed in methanol and acetic acid, incubated with phosphate buffer, α-amylase buffer, and periodic acid, before staining with Schiff’s reagent. Samples were washed thoroughly with dH_2_O between each step. Samples were mounted in chloral hydrate and imaged using a Zeiss LSM 780 confocal microscope (Carl Zeiss). Cell counting and cell length measurement were performed using ImageJ 1.48v (Cell-o-Tape plugin, 0.7.7v) (French *et al.*, 2012).

### Confocal laser scanning microscopy of Arabidopsis roots

Images of Col-0 lines expressing *pAtCNGC15:AtCNGC15:GFP* and *pAtUBI10:AtCNGC15*^*D408N*^*:GFP* for localization were collected using a Zeiss LSM780 confocal microscope with a ×40/1.2 water objective or a ×20/0.5 dry objective. The GFP excitation wavelength used was 488 nm, and emitted fluorescence was collected at 500–550 nm.

### Nodulation complementation assays

Assays were carried out as described in Leitão *et al.* (2019). In brief, the Golden Gate constructs *pAtUBI10:GFP* and *pAtUBI10:CNGC15*^*D408N*^*:GFP* were expressed in *M. truncatula* roots using *Agrobacterium rhizogenes*-mediated gene transfer performed as previously described (Leitão *et al.*, 2019). One-week-old plants were grown in Terragreen/Sand (Oil-Dri Company, Wisbech, UK) in a 1:1 ratio and inoculated with *Sinorhizobium meliloti* 2011 (OD_600_=0.01). Nodules were scored after 25 d.

### Calcium imaging

Ca^2+^ imaging was performed as described in Leitão *et al.* (2019). In brief, 5 days after germination (dag), seedlings were collected and positioned in a small chamber made with a coverslip and high-vacuum grease (Dow Corning GMBH, Wiesbaden, Germany). The chamber was filled with 50–100 µl MS medium and the whole root was covered with a smaller coverslip. The seedling was left at room temperature for around 45 min before imaging. Images of the root tip were collected every 2–3 s for an average of 1.5 h. Analysis was carried out using ImageJ 1.48v exactly as described in Leitão *et al.* (2019), using an algorithm designed to analyse the rise and fall times of the calcium spike.

### Modelling of the AtCNGC15 structure

The Protein Data Bank was interrogated using the amino acid sequence of AtCNGC15. This yielded several hits with ~80% coverage of the target sequence, with sequence identities in the range of 17–24%. Homology models were generated using the SWISS-MODEL server (Waterhouse *et al.*, 2018), which has the advantage of retaining the homotetrameric quaternary structure of the template. We focused on models generated from the structures of Eag1 from *Rattus norvegicus* (PDB code 5K7L; Whicher and MacKinnon, 2016), human hERG (PDB code 5VA1; Wang and MacKinnon, 2017) and TAX-4 from *Caenorhabditis elegans* (PDB code 5H3O; Mazzolini *et al.*, 2018). In all cases, D408 was located on the channel-facing side of helix Aʹ and adjacent to R398 at the junction of helices S6 and Aʹ in the neighbouring subunit.

Given the low sequence identities with known structures, we also generated template-free, *ab initio* predictions of the AtCNGC15 structure using AlphaFold2 (AF2) (Jumper *et al.*, 2021) as implemented in ColabFold (Mirdita *et al.*, 2022). The five independent models generated were very similar to one another and showed high quality local structure (pLDDT scores) across most of the sequence and, crucially, predicted a subunit–subunit interface with relatively high confidence (predicted aligned error scores) that was consistent with those observed in the SWISS-MODEL results. Furthermore, D408 and R398 from neighbouring subunits were adjacent to one another in all five AF2 models and the same salt-bridge linking the two subunits could be modelled. Structural figures were produced using ChimeraX (Pettersen *et al.*, 2021).

### Expression in *Xenopus laevis* oocytes

*AtCNGC15* cDNA was synthetized according to the codon usage of *X. laevis* by GenScript and subcloned using Gateway cloning (Katzen, 2007) into *pOO2-GW* (Chen *et al.*, 2010) for electrophysiology and *pGCS-C6(eGFP)* (Wang *et al.*, 2016) for localization. Site directed mutagenesis of the codon-optimized *AtCNGC15* was done using overlapping primers (Supplementary Table S1) to generate the *pOO2-GW-AtCNGC15*^*D408N*^ and *pGCS-C6(eGFP)-AtCNGC15*^*D408N*^ constructs (Liu and Naismith, 2008). *In vitro* transcription was performed using the mMESSAGE MMACHINE SP6 kit (Thermo Fisher Scientific), to yield capped and polyadenylated RNA that was purified using phenol–chloroform extraction (Merck). Oocytes were injected with 55 ng of cRNA in 50 nl of RNase-free water or with 50 nl of RNase-free water using a compressed air system. Injected oocytes were incubated at 17°C in modified Barth’s solution (MBS) pH 7.4 (88 mM NaCl, 1 mM KCl, 2.4 mM NaHCO_3_, 0.3 mM Ca(NO_3_)_2_, 0.41 mM CaCl_2_, 0.82 mM MgSO_4_, 15 mM Hepes) supplemented with 10 g ml^−1^ sodium penicillin and 10 µg ml^−1^ streptomycin sulphate. One day before electrophysiology, oocytes were transferred to a calcium-free MBS (same as the above except for the absence of Ca(NO_3_)_2_ and CaCl_2_).

### Electrophysiology

Whole oocyte currents and membrane potential were recorded using the two-electrode voltage-clamp technique 2 d after cRNA injection according to Charpentier *et al.* (2016) with the following modification: the equipment used was the Axoclamp 900A microelectrode amplifier and the Digidata 1440A digitizer (Molecular Devices), and voltage-pulse protocols, data acquisition, and analysis were performed using pCLAMP 10.7 software (Molecular Devices). Electrodes were filled with 3 M KCl. The oocytes were continuously perfused during the voltage-clamp experiment with either calcium-free MBS or calcium-free MBS supplemented with 20 mM CaCl_2_ or 20 mM BaCl_2_. Voltage steps were applied from −165 to +40 mV in +15 mV increments, and the currents were recorded to obtain current–voltage curves.

### Confocal laser scanning microscopy of *Xenopus* oocytes

Two days after injection with cRNA or H_2_O, images were taken using a Zeiss LSM780 microscope with ZEN 2012 SP5 (Black) acquisition software, a ×10/0.3 dry objective and the tiling function. A 25 mW argon ion laser was used with an excitation wavelength of 488 nm and emitted fluorescence was collected at 520 nm. Confocal pinhole size was 1 AU. The 32-channel GaAsP photomultiplier tube array was used with 200 V gain. Quantification of fluorescence was carried out in ImageJ. The area, integrated intensity, and mean grey values of each oocyte were measured and compared with that of the background to give the total fluorescence of each oocyte normalized to the background.

### Quantification of gene expression

RNA extraction from Arabidopsis root tips was performed with the RNeasy Plant Micro Kit (Qiagen) according to the manufacturer’s instructions. cDNA was obtained from 1000 ng of RNA using the SuperScript II Reverse Transcriptase (Thermo Fisher Scientific) as per the manufacturer’s instructions. Quantitative PCR (qPCR) was performed as described in Leitão *et al.* (2019). Calculation of expression levels was performed using a previously described mathematical equation (Pfaffl, 2001), adapted to account for the use of two reference genes as described in Vandesompele *et al.* (2002) and Hellemans *et al.* (2007):


$$\begin{array}{l} Relative\;Gene\;Expression\;=\;\frac{{(E_{GOI})}^{\Delta C_{t}\left(GOI\right)}}{GeoMean\left[{\left(E_{Ref1}\right)}^{\Delta C_{t}\left(Ref1\right)},{\left(E_{Ref2}\right)}^{\Delta C_{t}\left(Ref2\right)}\right]} \end{array}$$

where GeoMean is geometric mean, the *n*th root of the product of *n* numbers, and GOI is gene of interest.

Primers used for qPCR are listed in Supplementary Table S1.

### Nitrate treatment of Arabidopsis roots

Seedlings were grown on nitrate-free ½ MS plates as described above for 6 d. At day 6, seedlings were incubated in liquid nitrate-free ½ MS supplemented with either 10 mM KNO_3_ or 10 mM KCl (control treatment) for 30 min. The root tip samples were cut and frozen in liquid nitrogen prior to RNA extraction and qRT-PCR described above.

### Statistical analyses

Statistical analyses were performed using GraphPad Prism version 5.00 for Windows (GraphPad Software, San Diego, CA, USA).

## Results

### AtCNGC15 is required for root apical meristem development in young seedlings

**Fig. 1.**
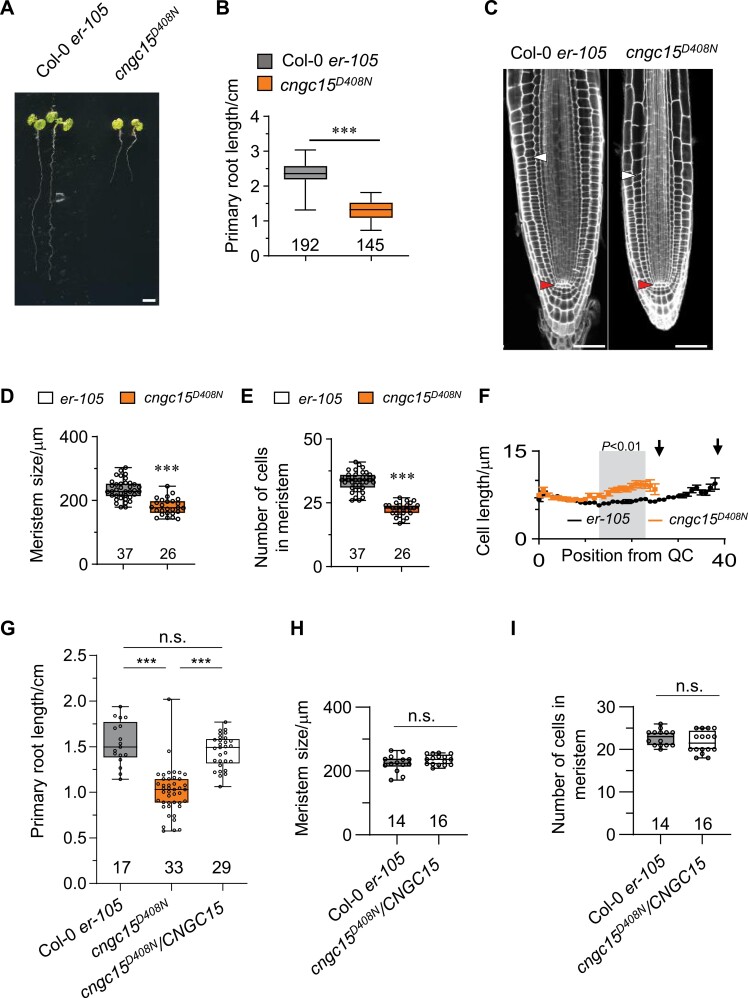
*cngc15* ^*D408N*^ is impaired in primary root development. (A) Representative image of Col-0 *er-105* and *Atcngc15*^*D408N*^ seedlings 6 days after germination (dag). Scale bar: 0.2 cm. (B) Primary root length of wild type (Col-0 *er-105*, *n*=192) and *Atcngc15*^*D408N*^ (*n*=145) at 6 dag. (C) Cellular organization of the root meristem visualized by confocal microscopy after staining with propidium iodide of wild type (Col-0 *er-105*) and *Atcngc15*^*D408N*^ at 6 dag. White and red arrowheads mark the first elongated cortex cell and the quiescent centre (QC), respectively. Scale bars: 50 µm. (D–F) Root meristem length (D), root meristem cell number (E), and cell length over cell position from the QC to the last meristematic cortex cell (F) of wild type (Col-0 *er-105*, *n*=37) and *Atcngc15*^*D408N*^ (*n*=26). Black arrows in (F) mark the last meristematic cell and areas shaded in grey indicate significant differences between the genotypes (*P*<0.01, two-tailed *t*-test with a prior *F*-test for homoscedasticity). (G) Primary root length in Col-0 *er-105* (*n*=17), *Atcngc15*^*D408N*^ (*n*=33), and *Atcngc15*^*D408N*^ complementation line (*Atcngc15*^*D408N*^*AtUBI10:gAtCNGC15:GFP*, *n*=29) at 6 dag. (H, I) Root meristem size (H) and root meristem cell number (I) in Col-0 *er-105* (*n*=14) and *Atcngc15*^*D408N*^ complementation line (*Atcngc15*^*D408N*^*AtUBI10:gAtCNGC15:GFP*, *n*=16) at 6 dag. Statistical significance was assessed using a two-tailed *t*-test with a prior *F*-test for homoscedasticity, ***P<0.001. Box and whisker plots show 25% and 75% percentiles, median, minimum, and maximum, with sample size denoted underneath the plots.

The functional and molecular characterization of neuronal heteromeric CNG channels has long been advanced by the study of missense mutations in highly conserved residues of single CNGC units, which cause channel heterocomplex misfunction and severe pathology (Biel and Michalakis, 2007). To assess the function of the AtCNGC15 during root apical meristem development, we identified, by searching the Arabidopsis TILLING line collection (Till *et al.*, 2003), an *Atcngc15* mutant allele in which *CNGC15* is mutated in a highly conserved aspartic acid in the C-linker domain (D408N) (Supplementary Fig. S1A–C). The D408N mutation does not impair the expression of *AtCNGC15*^*D408N*^ (Supplementary Fig. S1D) or the localization of AtCNGC15^D408N^ at the nuclear envelope in the root apical meristematic cells after 6 d of growth (Supplementary Fig. S2). However, expression of *AtCNGC15*^*D408N*^ in the *Mtcngc15b,c* double mutant cannot restore the symbiotic defect (Supplementary Fig. S3), which suggests that the D408N mutation impairs AtCNGC15 function. To assess the effect of this mutation in Arabidopsis primary root growth, we monitored the root length of both the *Atcngc15*^*D408N*^ mutant, and the segregating population generated by backcrossing *Atcngc15*^*D408N*^, at 6 dag (Fig. 1; Supplementary Fig. S4). The root length analyses revealed that the *Atcngc15*^*D408N*^ mutant has a shorter primary root due to a reduced number of meristematic cells, which results in the establishment of a shorter root meristem (Fig. 1A–F). It also revealed that the D408N mutation is semi-dominant, with the primary root length of the heterozygotes being shorter than the wild type and longer than the homozygous *Atcngc15*^*D408N*^ (Fig. 1A, B; Supplementary Fig. S4A). To confirm the semi-dominant effect of the D408N mutation, we analysed the primary root length phenotype of the wild-type line overexpressing *AtCNGC15*^*D408N*^ fused to GFP (Supplementary Fig. S4B). Expression of *AtCNGC15*^*D408N*^ is sufficient to significantly reduce the primary root length in the wild-type background (Supplementary Fig. S4B). Similarly, overexpressing the genomic sequence of *AtCNGC15* fused to GFP in the *cngc15*^*D408N*^ mutant allele significantly restores the root apical meristem and primary root length defect (Fig. 1G–I). Together, our results reveal that the nuclear localized AtCNGC15 regulates meristematic cell number and therefore root apical meristem development during seedling growth.

### AtCNGC15 is required for the generation of nuclear calcium release

**Fig. 2.**
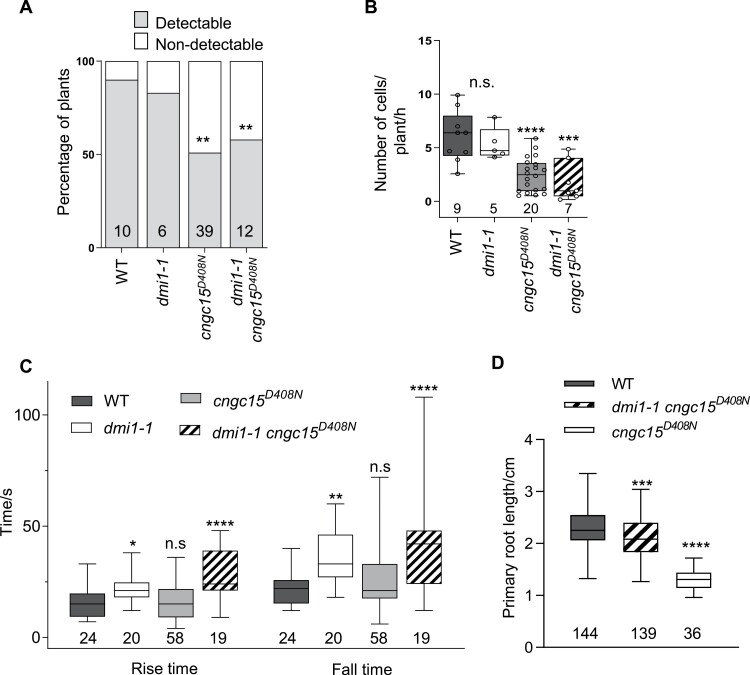
*Atcngc15* ^*D408N*^ impairs nuclear calcium release in the root apical meristem. (A) Percentage of plants that displayed cell autonomous nuclear Ca^2+^ spikes during root growth in wild type (WT), *dmi1-1*, *Atcngc15*^*D408N*^, and *dmi1-1Atcngc15*^*D408N*^ over 90 min of imaging. Numbers in bars represent the number of plants imaged (χ^2^-test), **P<0.01. (B) Number of cells releasing Ca^2+^ per plant per hour in WT, *dmi1-1*, *Atcngc15*^*D408N*^, and *dmi1-1Atcngc15*^*D408N*^ within 200 μm of meristem length. (C) Rise and fall times of the nuclear calcium spike recorded in WT, *dmi1-1*, *Atcngc15*^*D408N*^, and *dmi1-1Atcngc15*^*D408N*^. Rise time refers to the duration of the upward slope of the calcium spike, whilst fall time refers to the duration of the downward slope of the calcium spike. (D) Primary root length of WT, *Atcngc15*^*D408N*^, and *dmi1-1 Atcngc15*^*D408N*^ at 6 dag. (B–D) Statistical significance calculated using Dunnet’s multiple comparison one-way ANOVA to compare the mutant lines with WT, *P<0.05, **P<0.01, ***P<0.001, ****P<0.0001. The *n*-values are indicated below the box and whisker plots, which show 25% and 75% percentiles, median, minimum, and maximum.

Release of calcium inside the nucleoplasm of root apical meristem cells is associated with meristem development and the regulation of auxin homeostasis (Leitão *et al.*, 2019). We previously showed that the nuclear-localized AtDMI1 modulates this process, with AtDMI1 acting as a counter ion channel, balancing the efflux of calcium from the lumen of the nuclear envelope (Leitão *et al.*, 2019). Loss of *AtDMI1* in the *dmi1-1* mutant causes an increase in the duration of the nuclear calcium release and is associated with longer roots. In contrast, the overexpression of *AtDMI1* induced by a T-DNA insertion in its promoter (*Atdmi1-2*) impairs nuclear calcium release, leading to shorter roots (Leitão *et al.*, 2019). Reminiscent of the *Atcngc15*^*D408N*^ mutant phenotype, the short root phenotype of *Atdmi1-2* is due to a reduced number of meristematic cells, but whether AtCNGC15 modulates nuclear calcium release in the root apical meristem remains to be demonstrated. To test this hypothesis, we monitored the nuclear calcium release at 6 dag of wild type and *Atcngc15*^*D408N*^ mutant lines expressing a dual-localized fluorescent calcium sensor (nuclear-localized red and cytoplasmic-localized green GECO1.2; NRCG-GECO1.2) that allows simultaneous measurement of cytosolic and nuclear calcium releases (Kelner *et al.*, 2018). As with the mutant overexpressing *AtDMI1*, nuclear calcium release is impaired in *Atcngc15*^*D408N*^, with a significant reduction in the number of plants and meristematic cells in which nuclear calcium spikes can be detected over 90 min of recording (Fig. 2A–C). This result demonstrates that AtCNGC15 is required to modulate the nuclear calcium release associated with root apical meristem development.

In line with the previous study, our results support that reducing the frequency of meristematic cell spiking correlates with shorter primary roots, and also that increasing the duration of the nuclear calcium release is associated with longer roots (Leitão *et al.*, 2019). To further corroborate the relationship between nuclear calcium signatures and primary root development in Arabidopsis seedlings, we generated the double mutant *Atdmi1-1*/*cngc15*^*D408N*^ expressing NRCG-GECO1.2. Analyses of nuclear calcium spikes and primary root length phenotypes revealed that combining the two properties, a decrease in the frequency of nuclear spikes (Fig. 2A, B) and an increase in the mean duration of the nuclear spikes from 39.5 s to 57.1 s (Fig. 2C) is sufficient to significantly increase primary root length in comparison with *Atcngc15*^*D408N*^, but not restore it to wild-type levels (Fig. 2D). These results confirm that increasing the spike duration is sufficient to increase root length, but a normal frequency of spikes in the meristem is required to fully rescue the phenotype. This confirms that the nuclear calcium signature modulates root growth.

### The D408N point mutation in the C-linker impairs the gating properties of CNGC15

**Fig. 3.**
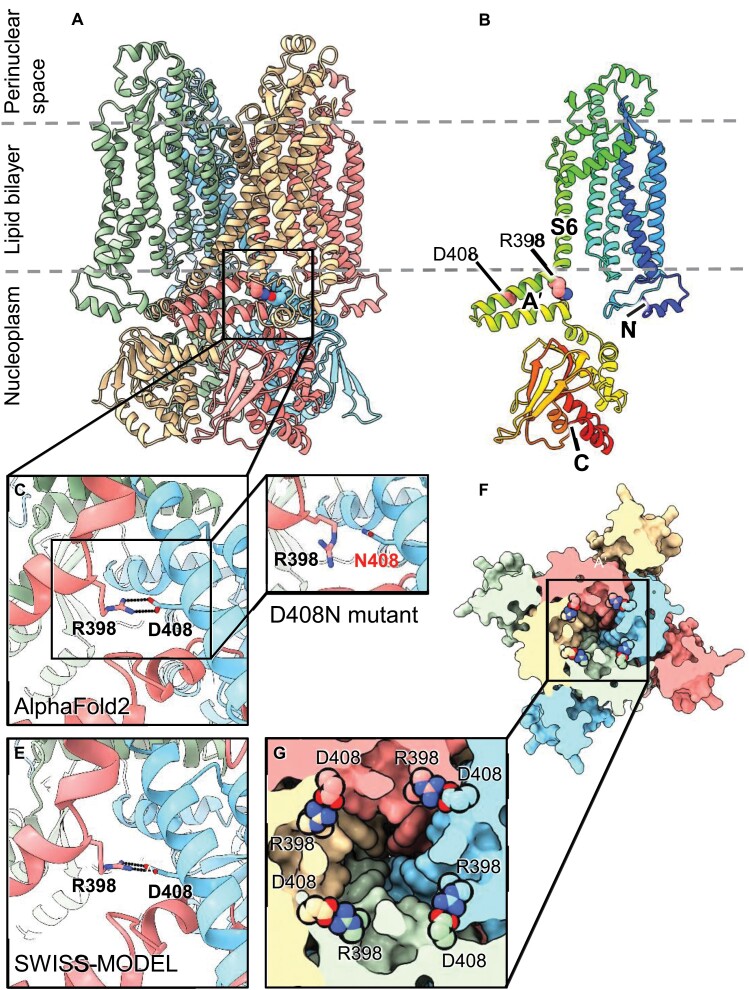
D408N mutation is predicted to disrupt salt bridges between adjacent AtCNGC15 subunits. (A) Cartoon representation of the AtCNGC15 model generated by AlphaFold2 (AF2), with the subunits illustrated in different colours. Poorly predicted regions at the N- and C-termini were deleted (residues 1–53 and 604–678, respectively). In the highlighted region, the residues contributing to one of the salt-bridges are shown as van der Waals spheres. (B) Single protomer corresponding to the salmon-coloured subunit in (A) illustrated with rainbow coloration from blue at the N-terminus through to red at the C-terminus. The positions of the residues D408 and R398 in the C-linker region referred to as the Aʹ helix are indicated. (C) Close-up of the region highlighted in (A) showing one of the four symmetry-related salt-bridges (for clarity the yellow subunit in the foreground of (A) is omitted). The inset (D) shows the loss of this potential salt-bridge in the D408N mutant. (E) Structurally equivalent region to that shown in (C) for the SWISS-MODEL homology model based on the Eag1 structure, illustrating the similarity to the AF2 model. (F) View of the AF2 model from the nucleoplasm (i.e. from below with respect to the view shown in (A)) with the structure depicted as a solid molecular surface that is cut away at the level of the salt-bridges. The latter are shown as van der Waals spheres. Note the location of the salt-bridges adjacent to the lumen of the channel. The inset (G) shows a close-up of this central region.

**Fig. 4.**
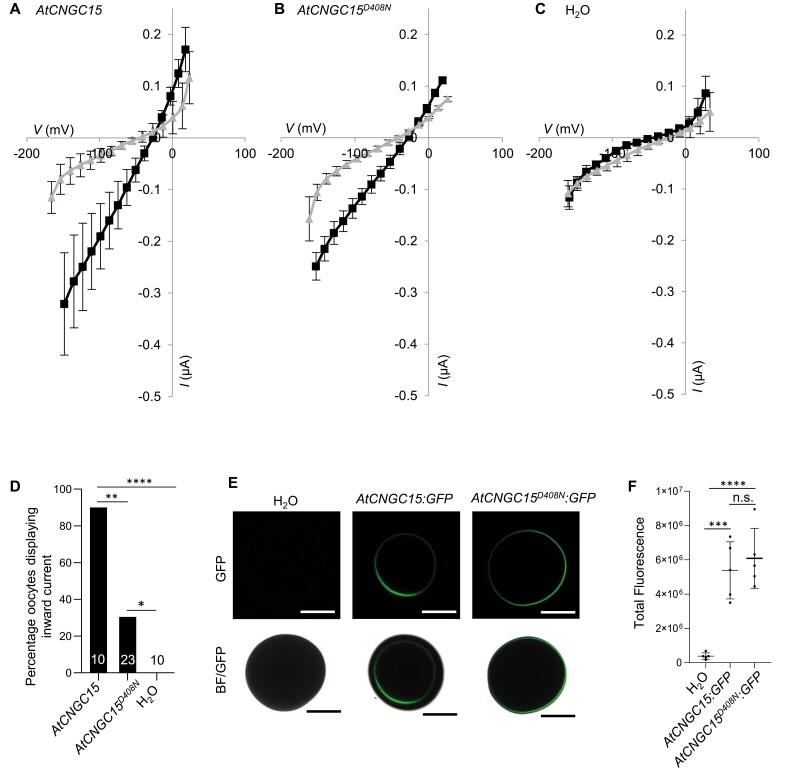
D408N mutation impairs the gating of CNGC15. (A–C), Average current–voltage (*I–V*) curves in oocytes expressing *AtCNGC15*, *AtCNGC15*^*D408N*^, or injected with water in the presence of 20 mM BaCl_2_ (grey) or 20 mM CaCl_2_ (black). Values represent means ±SD. (D) Percentage of oocytes displaying inward current upon calcium treatment when injected with At*CNGC15* or *AtCNGC15*^*D408N*^ cRNA, or water control. Statistical significance was assessed using two-tailed *t*-tests with a prior *F*-test for homoscedasticity (*P<0.05, **P<0.01, ****P<0.0001). Numbers in bars denote sample size. (E) Green fluorescence protein (GFP) fluorescence (upper panel) and merged GFP and bright field images (bottom panel) of *Xenopus* oocytes injected with H_2_O, *AtCNGC15-GFP* cRNA, or *AtCNGC15*^*D408N*^*-GFP* cRNA, taken with laser scanning confocal microscopy. Scale bars: 500 µm. (F) Quantified fluorescence intensity of *Xenopus* oocytes injected with H_2_O, *AtCNGC15-GFP* cRNA, or *AtCNGC15*^*D408N*^*-GFP* cRNA. Fluorescence of each oocyte was measured relative to the background. Box and whisker plots show 25% and 75% percentiles, median, minimum, and maximum. Statistical significance calculated using one-way ANOVA with Tukey’s multiple comparison post-hoc test (*n*=5, ***P<0.001, ****P<0.0001).

The phenotype of the *Atcngc15*^*D408N*^ mutant, combined with the semi-dominant nature of the D408N mutation, suggest that irrespective of whether AtCNGC15 functions as a homo- or heterocomplex, D408N is sufficient to impair calcium release. To further investigate the effect of the D408N mutation on AtCNGC15 function at the molecular level, we inspected structures predicted using AlphaFold2 and by homology modelling, which revealed that D408 is positioned in the C-linker region (Fig. 3A, B), known to modulate the opening and closing, also known as gating, of animal CNG channels (Mazzolini *et al.*, 2018). The D408 mutation is predicted to disrupt a salt bridge that forms at each interface, between D408 from one subunit and R398 of its neighbour, and thereby impact the gating properties of the channel (Fig. 3B–F). To test this hypothesis, we expressed *AtCNGC15* and *AtCNGC15*^*D408N*^ in oocytes of *Xenopus laevis* and recorded the channel activity using a two-electrode voltage clamp in the presence of 20 mM CaCl_2_ or 20 mM BaCl_2_ (Fig. 4A–C). Oocytes injected with *AtCNGC15* cRNA or *AtCNGC15*^*D408N*^ cRNA displayed a significant inward current in the presence of 20 mM CaCl_2_. In line with previous studies (Wang *et al.*, 2021), these results confirm that CNGC15 is a calcium channel and further ­demonstrate that the AtCNGC15^D408N^ channel is permeable to calcium. However, although the calcium current recorded in oocytes expressing *AtCNGC15*^*D408N*^ is comparable to that recorded in oocytes expressing *AtCNCG15*, the percentage of oocytes displaying inward calcium current was significantly reduced (Fig. 4D). Only 30% of oocytes injected with *AtCNCG15*^*D408N*^ presented open ion channels. To assess whether the D408N mutation would perturb the translation and/or localization of AtCNGC15^D408N^, we expressed either *AtCNGC15* or *AtCNGC15*^*D408N*^ fused to GFP in oocytes (Fig. 4E, F). The localization and the fluorescence intensity of AtCNGC15:GFP and AtCNGC15^D408N^:GFP are both identical (Fig. 4E, F), demonstrating that the absence of a calcium current in 70% of the oocyte recorded is not due to altered translation and/or mislocalization, but to the impaired opening of AtCNGC15^D408N^. However, once the mutant channel opens, a wild-type calcium current is produced. This observation is consistent with the *in planta* phenotype of *Atcngc15*^*D408N*^, which shows a reduced frequency of nuclear calcium release (Fig. 2A, B) but without altering the rise and fall time of the calcium signal (Fig. 2C). Together, these results demonstrate that the opening properties of AtCNGC15 are perturbed by the D408N mutation, which is predicted to weaken the interaction between the C-linker neighbouring units in the tetrameric CNGC15 complex. The gating mechanism of plant CNGCs is unknown, and these results support a role of the C-linker region in this gating, similarly to the animal counterpart CNG channels.

### The dual function of AtCNGC15 is promoted by high nitrate

**Fig. 5.**
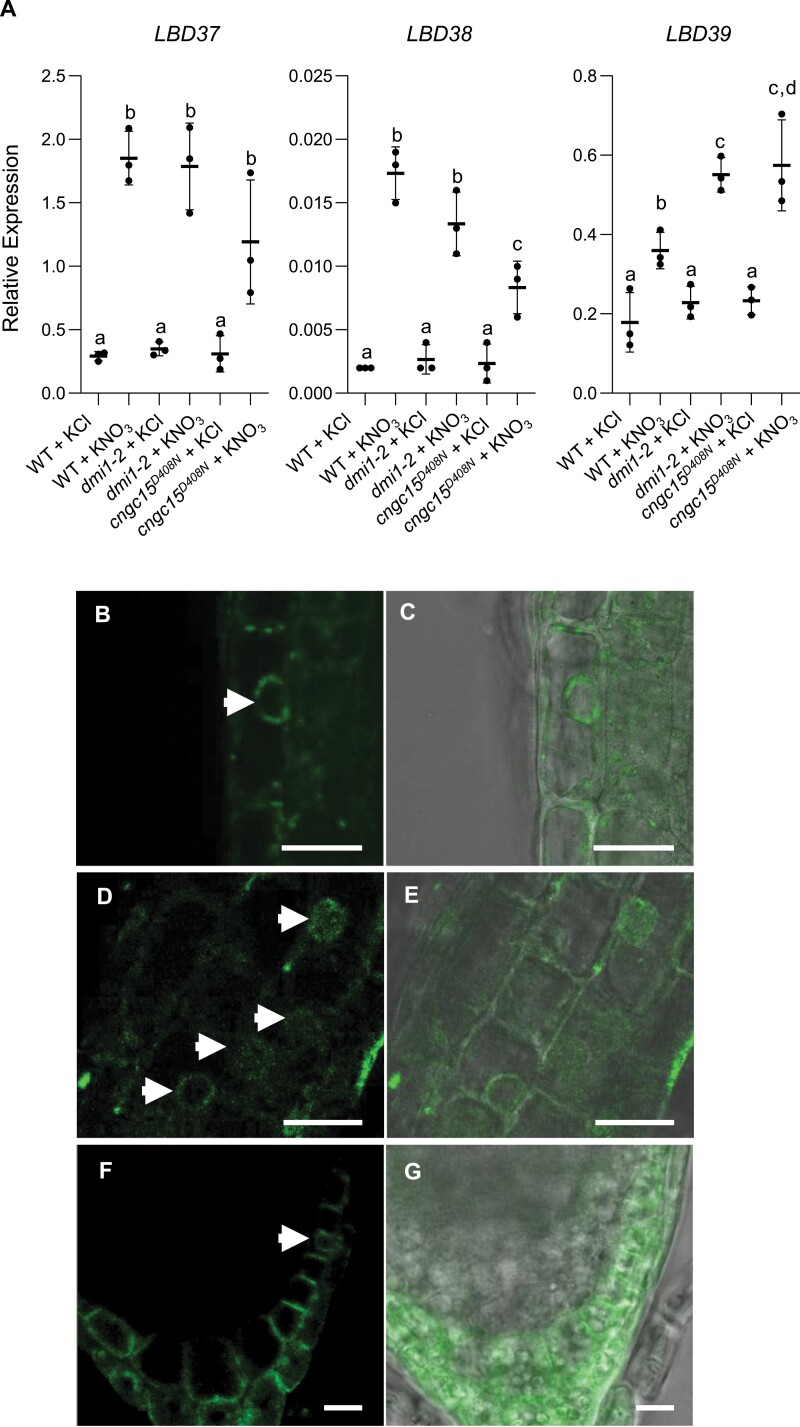
AtCNCG15 is required for nitrate-induced gene expression and relocalizes to the plasma membrane of columella cells upon high nitrate treatment. (A) Quantitative expression analyses of the transcript level of nitrate-regulated genes by qPCR in 6-day-old root tips of WT, *Atdmi1-2*, and *Atcngc15*^*D408N*^ seedlings (*n*=3, each sample is a pool of approximately 100 plants) on treatment with either 10 mM KCl or 10 mM KNO_3_ for 30 min. Expression was normalized to *UBIQUITIN 10* (*At4g05320*) and *UBOX* (*At5g15400*). Expression is displayed relative to these reference genes. Values are means ±SD. Letters represent statistical significance calculated using one-way ANOVA with Tukey’s multiple comparison post-hoc test. (B–G) Laser scanning confocal microscopy picture of a 6-day-old Arabidopsis root meristem expressing *pAtCNGC15:AtCNGC15:GFP*. Green fluorescent protein (GFP) detection (B, D, F) and overlay picture of the green channel and bright field (C, E, G) of the root meristem of seedlings grown in the presence of 10 mM nitrate. The arrows (B, D, F) indicate nuclear localization of CNGC15. Scale bars: 20 µm.

Primary nitrate sensing is modulated by the nitrate transceptor NRT1.1 (Ho *et al.*, 2009). NRT1.1 localizes at the plasma membrane of the outer cell layer of the root tip and root columella (Krouk *et al.*, 2010), where it is required for the nitrate-induced cytoplasmic calcium release that contributes to the expression of primary nitrate-responsive genes (Riveras *et al.*, 2015; Liu *et al.*, 2017). Notably, high nitrate causes nuclear translocation of the calcium-dependent protein kinase (CPK) 10, CPK30, and CPK36, which control the rapid induction of primary nitrate responsive genes, including the *LATERAL ORGAN BOUNDARIES DOMAIN* (*LBD*)*37*, *LBD38*, and *LBD39* (Liu *et al.*, 2017). Recent work demonstrated that AtCNGC15, which localizes at the nuclear envelope during root apical meristem establishment (Leitão *et al.*, 2019; Wang *et al.*, 2021), relocalizes to the plasma membrane at later stage (12 dag) of root growth, and associates with NRT1.1 to regulate the high nitrate-induced cytoplasmic calcium release and primary nitrate responsive gene expression (Wang *et al.*, 2021). This observation suggests that during early stages of root development (6 dag) the function of AtCNCG15 is distinct from nitrate signalling. To test this hypothesis, we quantified the expression of the nitrate-induced *LBD37*, *LBD38*, and *LBD39* (Liu *et al.*, 2017; Alvarez *et al.*, 2020) in 6-day-old root tips of wild type, *Atdmi1-2* and *Atcncg15*^*D408N*^ mutant lines, both impaired in nuclear calcium release, after a 30 min treatment with 10 mM KNO_3_ (Fig. 5A). Expression of *LBD37*, *LBD38*, and *LBD39* was significantly induced by nitrate in the wild type, demonstrating that primary nitrate signalling is unaffected at this developmental stage (Fig. 5A). Induction of *LBD37* was not affected in *Atdmi1-2* or *Atcngc15*^*D408N*^, indicating that *LBD37* expression is not impaired by alterations in nuclear calcium release (Fig. 5A). In contrast, the expression of the nitrate-induced *LBD39* was significantly enhanced in comparison with the wild type in both *Atdmi1-2* and *Atcngc15*^*D408N*^, suggesting that *LBD39* expression is negatively regulated by nuclear calcium release (Fig. 5A). Unexpectedly, induction of *LBD38* was significantly reduced in *Atcngc15*^*D408N*^ but not in *Atdmi1-2* root tips, suggesting, on one hand, that an impaired nuclear calcium release does not interfere with *LBD38* expression, and on the other hand, that AtCNGC15 might play a dual role at 6 dag in the presence of high nitrate. To test the possibility that a relocalization of AtCNGC15 to the plasma membrane was induced by nitrate, seedlings expressing *AtCNGC15:GFP* under its own promoter were grown on nitrate-free medium supplemented with either 10 mM KNO_3_ or 1 mM l-glutamine. Under both conditions, AtCNGC15 localized to the nuclear envelope in the root apical meristem after 6 d of growth (Fig. 5B–G; Supplementary Fig. S5). However, in the columella cells under high nitrate, AtCNGC15 localized to the plasma membrane as well as the nuclear envelope (Fig. 5F, G). This relocalization was not observed in the absence of nitrate (Supplementary Fig. S5), indicating that plasma membrane localization of AtCNGC15 at the columella cells is induced by nitrate treatment (Supplementary Fig. S5). This observation correlates with the expression of *NRT1.1*, which is highly expressed in, and localizes to, columella cells (Remans *et al.*, 2006; Krouk *et al.*, 2010). Together, our results indicate that in young seedlings, AtCNGC15 functions at the nuclear envelope to regulate nuclear calcium release that modulates both root development and nitrate-induced *LBD39* expression. However, upon high nitrate, AtCNGC15 relocalizes specifically to the plasma membrane of the columella cells where it contributes to the regulation of nitrate-induced *LBD38* expression. Our results further suggest that differential inputs of calcium are required to modulate *LBD37*, *LBD38*, and *LBD39* expression in response to nitrate.

## Discussion

During root apical meristem development, the observation that AtCNGC15 localizes to the nuclear envelope in proximity of AtDMI1 raised the question of whether AtCNGC15 contributes to the nuclear calcium release associated with root apical meristem growth in young seedlings (Leitão *et al.*, 2019; Wang *et al.*, 2021). In this study, we report the characterization of a novel *Atcncg15* mutant carrying a semi-dominant missense mutation in the highly conserved residue D408, positioned in the C-linker domain (Fig. 3; Supplementary Fig. S1). In line with a role of the C-linker in gating animal CNG channels, the D408N mutation is sufficient to impair the gating of AtCNGC15 in *X. laevis* oocytes and the frequency of nuclear calcium release in root apical meristem of *Atcngc15*^*D408N*^. This phenotype is also associated with shorter primary root meristems and root length (Figs 2, 4, 5). By combining the properties of the knock-out *Atdmi1-1* mutant allele (increased duration of the calcium release) with *Atcngc15*^*D408N*^ in the double mutant *Atdmi1-1Atcngc15*^*D408N*^, we modulated the nuclear calcium signal to increase its duration and decrease its frequency (Fig. 2). This is sufficient to partly restore the primary root length, confirming that the calcium specificity associated with root development is encoded in the frequency and duration of the nuclear calcium signal. Together our data show that AtCNCG15 modulates nuclear calcium release and root apical meristem development during early seedling growth, and further demonstrate that the C-linker is associated with the gating of CNGCs in plants. As the residue D408 is highly conserved among AtCNGCs (Supplementary Fig. S1B), it might represent a valuable target to develop new *AtCNGC* alleles and assess the effect of modulating the level of calcium release in CNGC-associated biological processes. Remarkably, although the D408N mutation is sufficient to impair the mechanism involved in opening AtCNGC15, the channel can still be opened, less readily, both *in planta* and in oocyte. This observation suggests that various mechanisms of activation might coexist, potentially including various ligands and/or cofactors whose effects can be influenced by the D408N mutation.

Trafficking of mammalian CNG and HCN channels to subcellular domains is critical for their multiple physiological roles (Lewis and Chetkovich, 2011). Interaction of HCN channels with auxiliary units, specific heterotetrametric assembly, and post-translational modifications of CNG channels are known to control ion channel trafficking (Trudeau and Zagotta, 2002; Han *et al.*, 2011). Recently, it was shown that at later stages of Arabidopsis root growth, overexpressed AtCNGC15 relocalizes to the plasma membrane of root cells where, in complex with NRT1.1, it contributes to the nitrate-induced cytoplasmic calcium release and gene expression changes (Wang *et al.*, 2021). The dual localization of AtCNGC15 was hypothesized to be developmental stage specific. As AtCNCG15 localizes to the nuclear envelope at 6 dag, this suggests that nitrate-induced gene expression in Arabidopsis root tips at 6 dag would be independent of AtCNGC15. Here, we demonstrate that the induction of the primary nitrate responsive genes, *LBD37*, *LBD38* and *LBD39*, occurs in 6-day-old Arabidopsis root tips. Interestingly, *LBD37*, *LBD38*, and *LBD39* are differentially regulated in the Arabidopsis mutants impaired for nuclear calcium release (*Atdmi1-2* and *Atcngc15*^*D408N*^) (Fig. 5A). The level of expression of *LBD39* is enhanced in comparison with the wild type in both mutants, while the expression of *LBD37* is similar, suggesting that nuclear calcium release negatively regulates expression of *LBD39* (Fig. 5A). In contrast, the nitrate induction of *LBD38* is only impaired in *Atcngc15*^*D408N*^. We also demonstrate that *AtCNGC15* expressed under its own promoter localizes to the nuclear envelope of the root meristem cells and relocalizes specifically in the columella cells to the plasma membrane in the presence of high nitrate (Fig. 5; Supplementary Fig. S5). Taken together we demonstrate that nuclear calcium mediated by AtCNGC15 can modulate nitrate-induced *LBD39* expression and root development. However, we show that in addition to regulating nuclear calcium signal in young seedlings, relocalization of AtCNGC15 at the plasma membrane specifically in columella cells can be induced by nitrate treatment where it contributes to regulating nitrate-induced *LBD38* expression. The differential regulation of the nitrate-induced *LBD37/38/39* in *Atdmi1-2* and *Atcngc15*^*D408N*^ highlights that the regulation of *LBD37*, *LBD38*, and *LBD39* expression requires different inputs of cytoplasmic and/or nuclear calcium.

The expression of *LBD37/38/39* is dependent on the activity of the calcium-regulated CPK10, CPK30, and CPK36, which were shown to relocalize to the nucleus upon nitrate treatment (Liu *et al.*, 2017). These data and our findings raise the question of whether the activities of CPK10, CPK30, and CPK36 require different amounts of cytoplasmic and/or nuclear calcium in a cell-specific manner to modulate nitrate-induced gene expression. The identification of *Atcngc15* mutant alleles impaired in gating, such as *Atcncg15*^*D408N*^, represents a valuable tool to assess *in planta* the connection between calcium input and specific activation of calcium decoders for future work.

## Supplementary data

The following supplementary data are available at *JXB* online. 

Fig. S1. Identification of the mutant allele *Atcngc15*^*D408N*^.

Fig. S2. *Atcngc15*^*D408N*^ localizes to the nuclear envelope.

Fig. S3. *Atcngc15*^*D408N*^ does not rescue the symbiotic phenotype of *M. truncatula cngc15b-2 cngc15c-1* double mutant.

Fig. S4. The mutation D408N encoded by *cngc15*^*D408N*^ is semi-dominant.

Fig. S5. AtCNCG15 does not localize to the plasma membrane in absence of nitrate.

Table S1. Primers used in this study.

Table S2. Golden Gate constructs.

Table S3. List of sequences used for the multiple sequence alignment.

erad041_suppl_Supplementary_Figures_S1-S5_Table_S1-S3

## Data Availability

All data supporting the findings of this study are available within the paper and within its supplementary materials published online.   The materials from this study are available from the corresponding author (MC), upon request.
