# Supplementary material for: A novel mutant allele of AtCNGC15 reveals a dual function of nuclear calcium release in the root meristem
Source: J Exp Bot. 2023 Jan 30;74(8):2572–84. doi: 10.1093/jxb/erad041 (PMC10112680; doi:10.1093/jxb/erad041)
Supplement: erad041_suppl_Supplementary_Figures_S1-S5_Table_S1-S3 [file erad041_suppl_supplementary_figures_s1-s5_table_s1-s3.pdf]

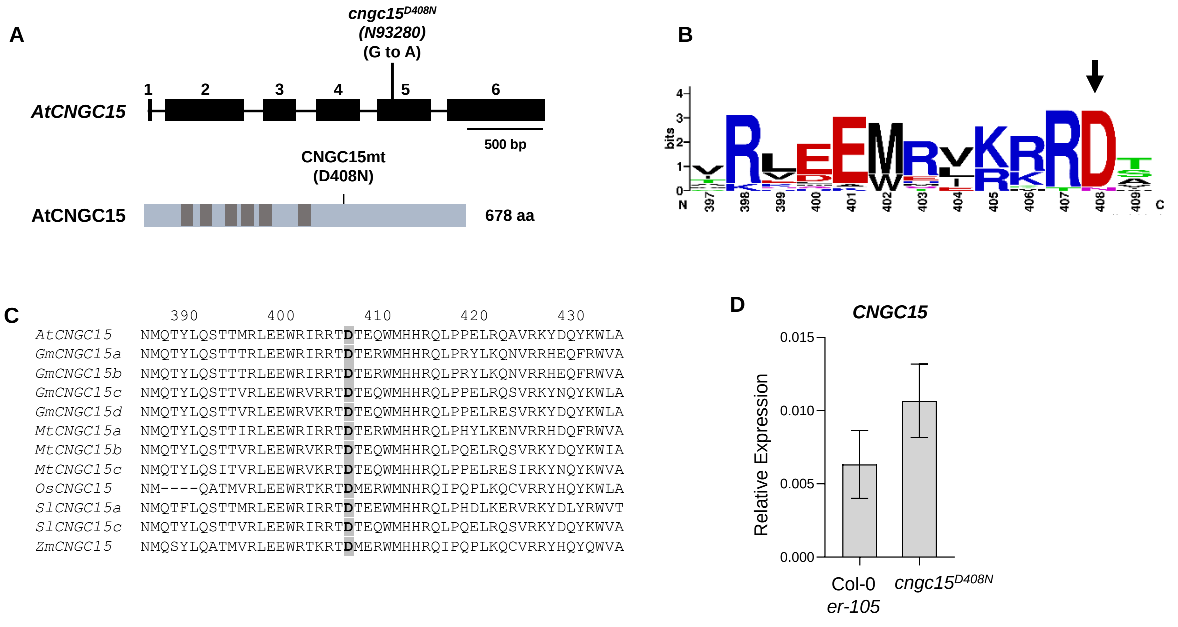

Fig. S1. Identification of the mutant allele *Atcngc15*^{D408N}.

A, Top: Position of the non-synonymous G to A point mutation in the genomic sequence of *AtCNGC15*. The 6 exons are numbered and represented by black boxes and introns by black lines. Bottom: Representation of amino acid sequence of AtCNGC15 including the position of the amino acid substitution in CNGC15^{D408N}. The transmembrane domains are shown in grey. B, Graphical representation of the multiple alignment of the amino acid sequences surrounding the conserved aspartic acid (D) 408 residues (marked by an arrow) in the 20 AtCNGCs (TAIR) via WebLogo (http://weblogo.threeplusone.com). C, Multiple sequence alignment using a Blosum62 cost matrix of the amino acid sequences of CNGC15 across multiple species (*Gm*, *Glycine max*; *Mt*, *Medicago truncatula; Os*, *Oryza sativa; Sl, Solanum lycopersicum*; *Zm*, *Zea mays*). The sequences used are presented in Table S3. Numbers indicate the amino acid position from the first, using the Arabidopsis sequence as a reference. The highly conserved aspartic acid D408 is indicated in the grey box. D, Quantitative expression analysis of the transcript level of *AtCNGC15* by RT-qPCR in root samples 6 days after germination (n = 3, pools of approximately 100 plants). Expression is displayed relative to the reference genes *UBOX* (*At5g15400*) and *UBIQUITIN 10* (*At4g05320*). Values are means ± SD (two-tailed t-test with a prior F-test for homoscedasticity).

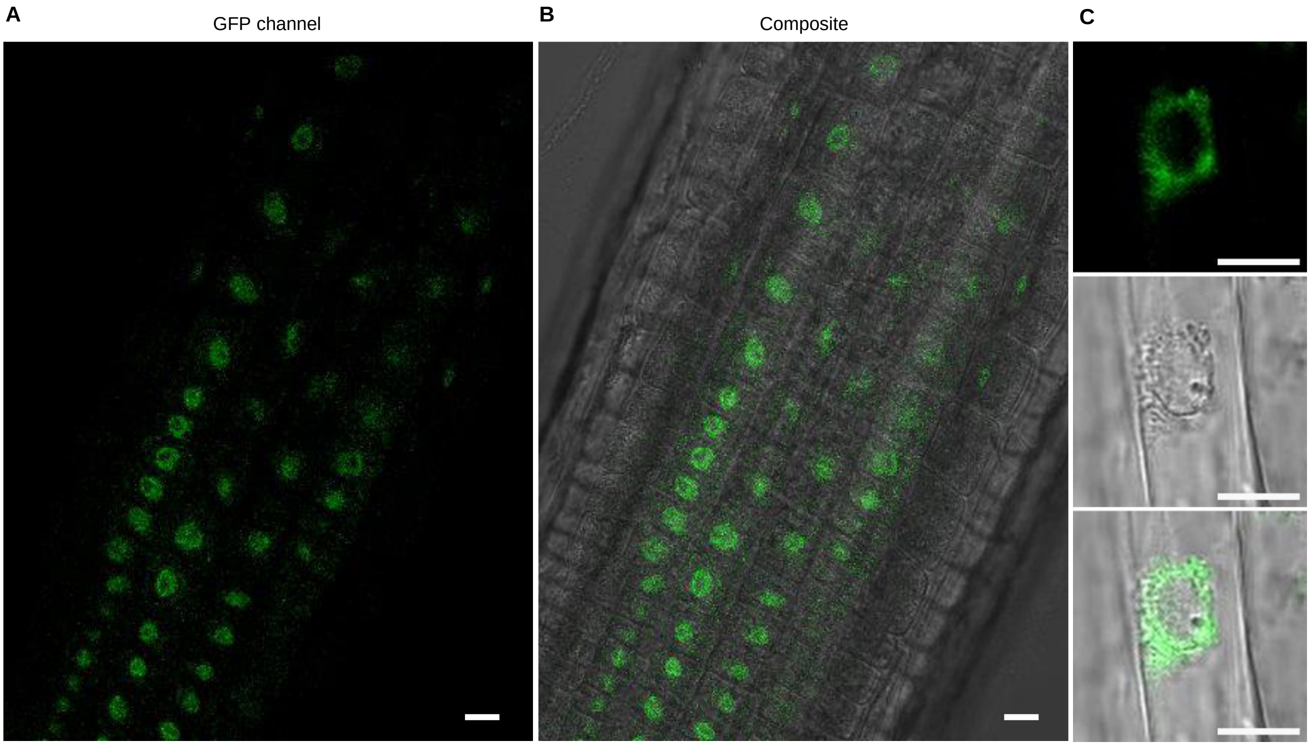

Fig. S2. *Atcngc15^{D408N}* localizes to the nuclear envelope.

Laser scanning confocal microscopy picture of a 6 day-old *A. thaliana* root meristem expressing *pAtUBI10::Atcngc15^{D408N}:GFP.* Green fluorescent protein (GFP) detection A, and overlay picture of the green channel and bright field, B. C, Zoom on one cell in the transition zone with Green fluorescent protein (GFP) detection (upper panel), bright field (middle panel), and overlay picture of the green channel and bright field (lower panel). Scale bars represent 10 µm.

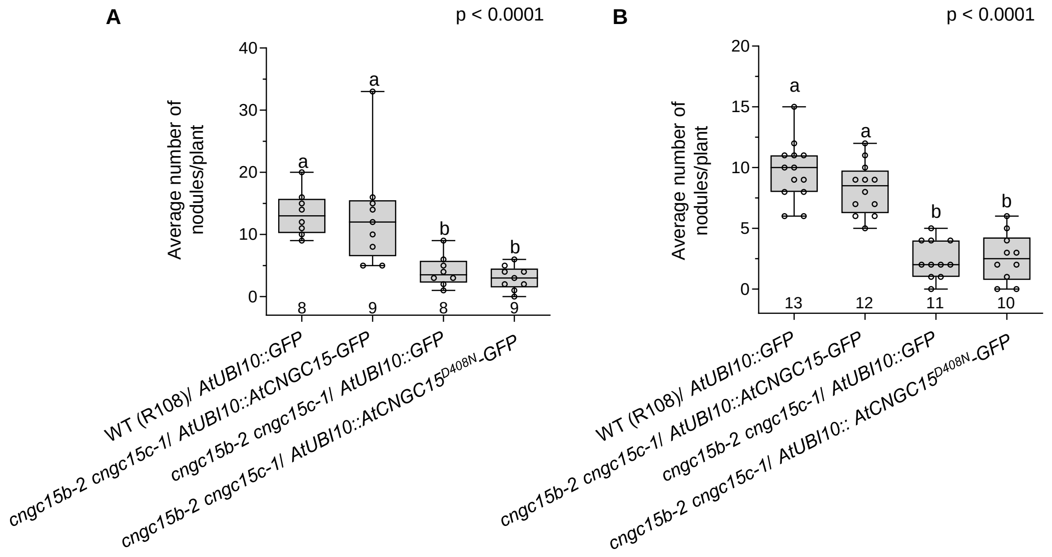

Fig. S3. *Atcngc15^{D408N}* does not rescue the symbiotic phenotype of *M. truncatula cngc15b-2 cngc15c-1* double mutant.

(A, B) Average number of nodules in *M. truncatula cngc15b-2 cngc15c-1* double mutant and wild type (WT) plants, whose roots have been transformed via *Agrobacterium rhizogenes* mediated transformation to express *AtCNGC15^{D408N}* fused to GFP, or GFP alone as the negative control. Expression was driven by the Arabidopsis *UBIQUITIN10* promoter. Each plant is an independent transformation event. Nodulation was assayed 25 days after inoculation with *S. meliloti* strain 2011. A and B represent two independent nodulation assays. Box and whisker plots show 25% and 75% percentiles, median, minimum and maximum. Numbers below box and whisker plots denote number of plants (sample size, n). Different letters indicate statistical differences and p values are inscribed (one-way ANOVA with Tukey's multiple comparison post-test).

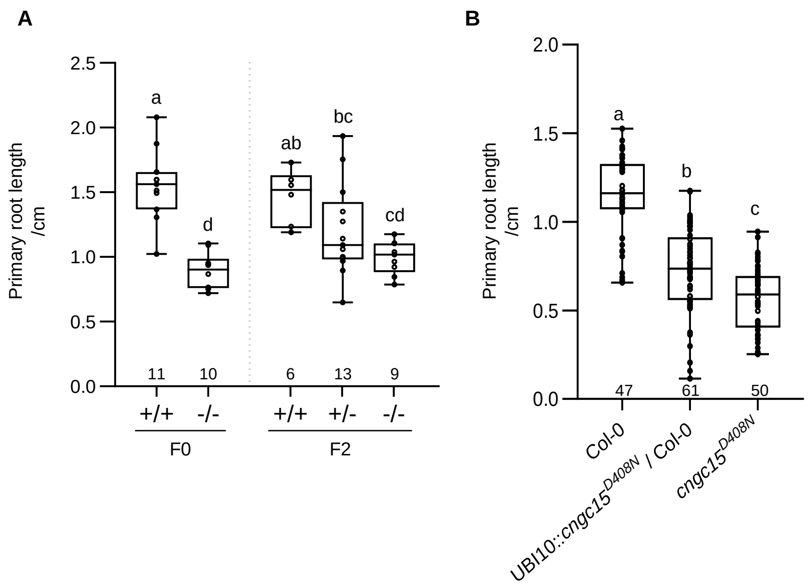

Fig. S4. The mutation D408N encoded by *cngc15^{D408N}* is semi-dominant.

A, Root length analyses six days after germination of the parent lines (F0) wild type (+/+) and mutant allele *cngc15^{D408N}* (-/-) as well as root length analyses of 28 F2 plants, the progeny of F1 plants generated by crossing F0 parents. +/+ indicates homozygous wild type, +/- indicates heterozygous for the *Atcngc15^{D408N}* mutation, and -/- indicates homozygous for the *Atcngc15^{D408N}* mutation. B, Primary root length analyses 6 dag of wild type ecotype Columbia, wild type over expressing *Atcngc15^{D408N},* and the mutant allele *Atcngc15^{D408N}.* Experiment was performed in three independent replicates. Box and whisker plots show 25% and 75% percentiles, median, minimum and maximum. A-B, Numbers below box plots denote sample size. Different letters indicate statistical differences (p-value<0.05 (A) and p-value<0.0005 (B); one-way ANOVA, Tukey's multiple comparisons test).

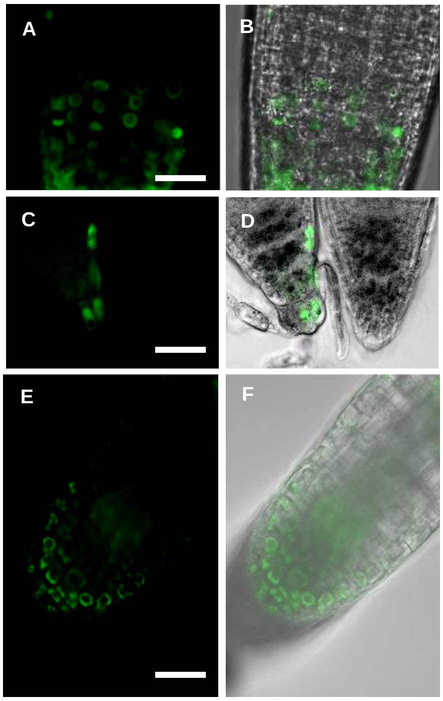

Fig. S5. AtCNCG15 does not localize to the plasma membrane in absence of nitrate.

A-F, Laser scanning confocal microscopy pictures of 6 day-old *A. thaliana* root meristems expressing *pAtCNGC15:AtCNGC15:GFP.* Green fluorescent protein (GFP) detection (A,C,E), and overlay picture of the green channel and bright field (B,D,F) of the root meristem of seedlings grown in the presence of 1 mM L-Glutamine as the nitrogen source (H-M). (C,D) Left seedling expresses *pAtCNGC15:AtCNGC15:GFP* and right seedling is a non-transformed seedling as a control. Scale bars represent 50 µm.

Table S1. Primers used in this study

Figure	FUNCTION	Sequence annealed	Name	Sequence (5'-3')		Efficiency (E)
S1	Genotyping	T-DNA (SALK)	LbB1.3	ATTTTGCCGATTTCGGAAC	LB	
S1	Genotyping	T-DNA (SAIL)	LB3	TAGCATCTGAATTTCATAACCAATCTCGATACAC	LB	
S1	Genotyping	*cngc15-1*	5	CAATATGCAAACCAGCAAGCAAG	fwd	
S1	Genotyping	*cngc15-1*	6	TTAACCGGGTCGCCTTCTCTTACAA	rev	
S5	Genotyping	*dmi1-1*	7	CAGAGTTTGCATGGAACAATG	LP	
S5	Genotyping	*dmi1-1*	8	TGTGGTTGTGTTAGCAGAACG	RP	
S1	RT-qPCR	*CNGC15*	CNGC15fwd	CAAGACTGAGCTGAGGGCAA	fwd	100.8% (T_a = 56 ºC)
S1	RT-qPCR	*CNGC15*	CNGC15rev	CCACCATTCACTGCTAGCCT	rev	
S1	RT-qPCR	*UBOX*	UBOXfwd	TGCGCTGCCAGATAATACACTATT	fwd	100.1% (T_a = 58 ºC)
S1	RT-qPCR	*UBOX*	UBOXrev	TGCTGCCCAACATCAGGTT	rev	
5	RT-qPCR	*LBD37*	LBD37fwd	TTGGACCGGCGAATTAACAAC	fwd	98.9% (T_a = 60 ºC)
5	RT-qPCR	*LBD37*	LBD37rev	AACCGAGAGAACAAAACGACG	rev	
5	RT-qPCR	*LBD38*	LBD38fwd	AGGAGGAGCAACGACAAAGTT	fwd	100.14% (T_a = 60 ºC)
5	RT-qPCR	*LBD38*	LBD38rev	GCCGTTCAAGCGAAGAGATTG	rev	
5	RT-qPCR	*LBD39*	LBD39fwd	TGGTTAAGGGTGATCGTCCG	fwd	100.07% (T_a = 60 ºC)
5	RT-qPCR	*LBD39*	LBD39rev	GATTCCTCCGATGGACTTCCC	rev	
5	qRT-PCR	*UBIQUITIN10*	At-UBIQUITIN-F	GGCTTGTATAATCCCTGATGAATAAG	fwd	93.3% (T_a=60ºC)
5	qRT-PCR	*UBIQUITIN10*	At-UBIQUITIN-R	AAAGAGATAACAGGAACGAAACATAGT	rev	
4	Site-directed mutagenesis	Codon optimized *AtCNGC15*	AtCNGC15-SDM-D408N-F	GAAGGACTAATACAGAACAGTGGATGC	fwd	
4	Site-directed mutagenesis	Codon optimized *AtCNGC15*	AtCNGC15-SDM-D408N-R	GTTCTGTATTAGTCCTTCTAATCCTCCAC	rev	

Table S2. Golden Gate constructs

Figure	Construct		
	position 1	position 2	position 3
Fig. S3	pNOS:BAR:tNOS	pAtUBI10:GFP:T35S	pATUBI10:NESmcherry:T35S
Fig. S3	pNOS:BAR:tNOS	pAtUBI10:gAtCNGC15:GFP:T35S	pATUBI10:NESmcherry:T35S
Fig. 1	pNOS:BAR:tNOS	pAtUBI10:gAtCNGC15:GFP:T35S	
Fig. S3	pNOS:BAR:tNOS	pAtUBI10:gAtCNGC15^{D408N}:GFP:T35S	pATUBI10:NESmcherry:T35S
Fig. S2	pNOS:BAR:tNOS	pAtUBI10:gAtCNGC15^{D408N}:GFP:T35S	
Fig. S5 & Fig. 5	pNOS:BAR:tNOS	pAtCNGC15:gAtCNGC15:GFP:T35S	

Table S3. List of sequences used for the Multiple sequence alignment (Fig. S1)

Names used in Extended Figure 5	Unique identifier	Organism	Source
AtCNGC15	AT2G28260.1	Arabidopsis thaliana	TAIR
GmCNGC15a	GLYMA13G20420.2	Glycine max	Phytozome
GmCNGC15b	GLYMA10G06121.1	Glycine max	Phytozome
GmCNGC15c	GLYMA12G08160.1	Glycine max	Phytozome
GmCNGC15d	GLYMA12G29840.1	Glycine max	Phytozome
MtCNGC15a	Medtr1g064240.1	Medicago truncatula	Phytozome
MtCNGC15b	Medtr4g058730.1	Medicago truncatula	Phytozome
MtCNGC15c	Medtr2g094860.1	Medicago truncatula	Phytozome
OsCNGC15	LOC_Os02g41710.1	Oryza sativa var. japonica	http://www.biomedcentral.com/1471-2164/15/853/abstract
SlCNGC15a	Solyc09g007840.2_1	Solanum lycopersicum	Phytozome
SlCNGC15c	Solyc11g069580.1_1	Solanum lycopersicum	Phytozome
ZmCNGC15	GRMZM2G068904_P01	Zea mays	Phytozome
